# Contusion of Penis by Water

**Published:** 1918-08

**Authors:** 


**Contusion of Penis by Water.** Peyre, Jour. de Med. de Bordeaux, Dec. 5, 1917, reports a workman subjected to a pressure of 150,000 kilos. There was rupture laterally, an enormous haematoma and haematuria developed. The outcome is not stated.

